# Gravid oviposition sticky trap and dengue non-structural 1 antigen test for early surveillance of dengue in multi-storey dwellings: study protocol of a cluster randomized controlled trial

**DOI:** 10.1186/s40249-019-0584-y

**Published:** 2019-09-03

**Authors:** Jonathan Wee Kent Liew, Sivaneswari Selvarajoo, Wing Tan, Rafdzah Ahmad Zaki, Indra Vythilingam

**Affiliations:** 1 Department of Parasitology, Faculty of Medicine, University of Malaya, 50603 Kuala Lumpur, Malaysia; 2 Centre for Epidemiology and Evidence Based Practice, Department of Social and Preventive Medicine, Faculty of Medicine, University of Malaya, 50603 Kuala Lumpur, Malaysia

**Keywords:** *Aedes*, Mosquito, Dengue, Dengue NS1 test, Gravid oviposition sticky trap, Cluster randomized controlled trial, Surveillance

## Abstract

**Background:**

Dengue is a global disease, transmitted by the *Aedes* vectors. In 2018, there were 80 615 dengue cases with 147 deaths in Malaysia. Currently, the nationwide surveillance programs are dependent on *Aedes* larval surveys and notifications of lab-confirmed human infections. The existing, reactive programs appear to lack sensitivity and proactivity. More efficient dengue vector surveillance/control methods are needed.

**Methods:**

A parallel, cluster, randomized controlled, interventional trial is being conducted for 18 months in Damansara Damai, Selangor, Malaysia, to determine the efficacy of using gravid oviposition sticky (GOS) trap and dengue non-structural 1 (NS1) antigen test for early surveillance of dengue among *Aedes* mosquitoes to reduce dengue outbreaks. Eight residential apartments were randomly assigned into intervention and control arms. GOS traps are set at the apartments to collect *Aedes* weekly, following which dengue NS1 antigen is detected in these mosquitoes. When a dengue-positive mosquito is detected, the community will be advised to execute vector search-and-destroy and protective measures. The primary outcome concerns the the percentage change in the (i) number of dengue cases and (ii) durations of dengue outbreaks. Whereas other outcome measures include the change in density threshold of *Aedes* and changes in dengue-related knowledge, attitude and practice among cluster inhabitants.

**Discussion:**

This is a proactive and early dengue surveillance in the mosquito vector that does not rely on notification of dengue cases. Surveillance using the GOS traps should be able to efficiently provide sufficient coverage for multistorey dwellings where population per unit area is likely to be higher. Furthermore, trapping dengue-infected mosquitoes using the GOS trap, helps to halt the dengue transmission carried by the mosquito. It is envisaged that the results of this randomized controlled trial will provide a new proactive, cheap and targeted surveillance tool for the prevention and control of dengue outbreaks.

**Trial registration:**

This is a parallel-cluster, randomized controlled, interventional trial, registered at ClinicalTrials.gov (ID: NCT03799237), on 8th January 2019 (retrospectively registered).

**Electronic supplementary material:**

The online version of this article (10.1186/s40249-019-0584-y) contains supplementary material, which is available to authorized users.

## Multilingual abstracts

Please see Additional file 1 for translations of the abstract into the five official working languages of the United Nations.

## Background

Dengue is a major mosquito-borne viral disease worldwide, especially in the tropical and sub-tropical countries. *Aedes* mosquitoes (mainly *Ae. aegypti* and *Ae. albopictus*) are the vectors of the disease. Globally, some 390 million dengue cases occur annually, 96 million of which are clinically apparent [1]. In Malaysia, a total of 80 615 cases of dengue with 147 deaths were reported in 2018. During the same year, the state of Selangor reported the highest number of cases (45 349 cases) and deaths (41 deaths) [2]. Subsequently, within the first ten weeks of 2019, Selangor accounted for more than half (16748) of the 28 936 dengue cases in Malaysia [3].

At present, anti-dengue drugs are not available, while dengue vaccine is not efficacious enough to act as a standalone intervention [4]. Additionally, the current vector surveillance/control measures – which are the hallmark of dengue control programs in many Southeast Asian nations, including Malaysia [5], usually involve house-to-house larval surveys, source reductions, larviciding, and fogging, all of which are riddled with downsides like non-relevance [6], cost- and labor-intensiveness [7], lack of community participation, as well as resistance of mosquitoes to insecticides [8–11]. The fact that asymptomatic, dengue-infected individuals are able to spread the disease has also been largely ignored [12]. These are the likely reasons for the alarming rise in dengue epidemics and the struggle to control them. Accordingly, proactive and sensitive methods which facilitate the early detection of dengue are desperately needed to pre-empt dengue outbreaks.

Besides the mentioned flaws of the dengue control program, the correlations of larval indices/adult mosquito emergence with dengue cases and the proportion of people who seek medical care post-infection are poor [6]. Furthermore, due to the passive nature of the current surveillance system, vector control activities mainly prevent further transmission of dengue from index cases rather than eliminate the disease. Therefore, the existing, reactive programs lack sensitivity and proactivity, and hence lack the ability to curb dengue epidemics. In view of all these shortcomings, more sensitive and proactive methods involving the early detection of dengue that can potentially stave off dengue outbreaks are desperately needed.

Although novel techniques, such as the release of genetically-modified mosquitoes (release of insects carrying dominant lethality; RIDL) and the use of *Wolbachia* bacteria to control *Ae. aegypti* populations, have great potential, many of such interventions/products are still under trial [13–17]. Urgent and effective strategies for vector surveillance/control are required pending the results of the aforementioned trials, since their assessments are highly time-consuming. In this matter, community support must also be sought. Ultimately, regardless of whether these novel techniques will eventually be implemented, vector surveillance remains an indispensable component of dengue control. Vector surveillance should be routinely and thoroughly conducted to prevent dengue epidemics. The World Health Organization (WHO) has called for the development of integrated national vector surveillance and health information systems to guide vector control measures [18].

Many traps of different designs have been tested for mosquito surveillance [19–21] and the use of sticky traps to lure and trap gravid adult *Aedes* females for vector surveillance/control appears to be promising in a number of countries [22–28]. Moreover, dengue non-structural antigen 1 (NS1) rapid test is a simple and reliable tool for detecting dengue in mosquitoes caught by the sticky traps [27, 29]. In a preliminary study conducted in an urban area in Selangor, Malaysia, infected *Ae. aegypti* mosquitoes could be obtained from sticky non-insecticidal gravid oviposition sticky (GOS) traps [26]. The use of the GOS traps and dengue NS1 rapid test kits allowed the rapid detection of dengue in the trapped mosquitoes, unlike the more sophisticated reverse-transcription PCR (RT-PCR). Accordingly, the health personnel involved in the study have found both instruments to be an easier method of dengue surveillance rather than labor-intensive larval surveys. Subsequently a prospective, longitudinal phase 2 study found dengue cases to occur around one week following the detection of infected mosquitoes, with a peak lag of 2–3 weeks [27]. Thus, this method of surveillance is likely to be feasible for incorporation into dengue control programs. Also, control methods such as search-and-destroy measures, should be initiated whenever positive mosquitoes are detected. In short, GOS traps and rapid dengue NS1 detection in mosquitoes offer a potentially comprehensive, early-warning surveillance system that is capable of pre-empting the occurrence of dengue epidemics.

Hence, the current phase of the research is aimed to determine if this adult *Aedes* surveillance/control method (using GOS traps and dengue NS1 antigen test) can actually reduce the occurrence of dengue outbreaks. The proposed novel approach entails the active monitoring of adult *Aedes* populations using easily constructed, inexpensive, reusable traps as well as rapid dengue-diagnostic kits. Vector surveillance is required to detect changes in vector abundance, evaluate control programmes, obtain spatial and temporal information of vector populations, as well as facilitate timely evidence-based interventions. In resource-limited settings, vector control/surveillance practices must be both efficient and efficacious [30]. If this paradigm is proven to be effective, a very viable alternative for curbing dengue will be made available not only to Malaysia, but to other low-to-middle-income countries as well.

## Design

### Aims

This parallel-cluster randomized controlled trial is designed with the aim of measuring the efficacy of a new, proactive paradigm (i.e. GOS traps and dengue NS1 rapid tests) to (i) reduce the occurrence of dengue cases vis-à-vis conventional vector surveillance measures; (ii) provide the community with early information of dengue transmission in the area, which will in turn provide more time for concise mitigative plans to be administered onto the community by public health authorities; as well as (iii) be workable and well-received by the management and residents of the housing areas. If its efficacy and practicability are proven, efforts will be undertaken to effectuate major revamps to the existing standard operating procedures through the Ministry of Health Malaysia and other policy makers. Additionally, a dossier will be submitted to the WHO for adoption by Southeast Asian countries.

**Fig. 1:**
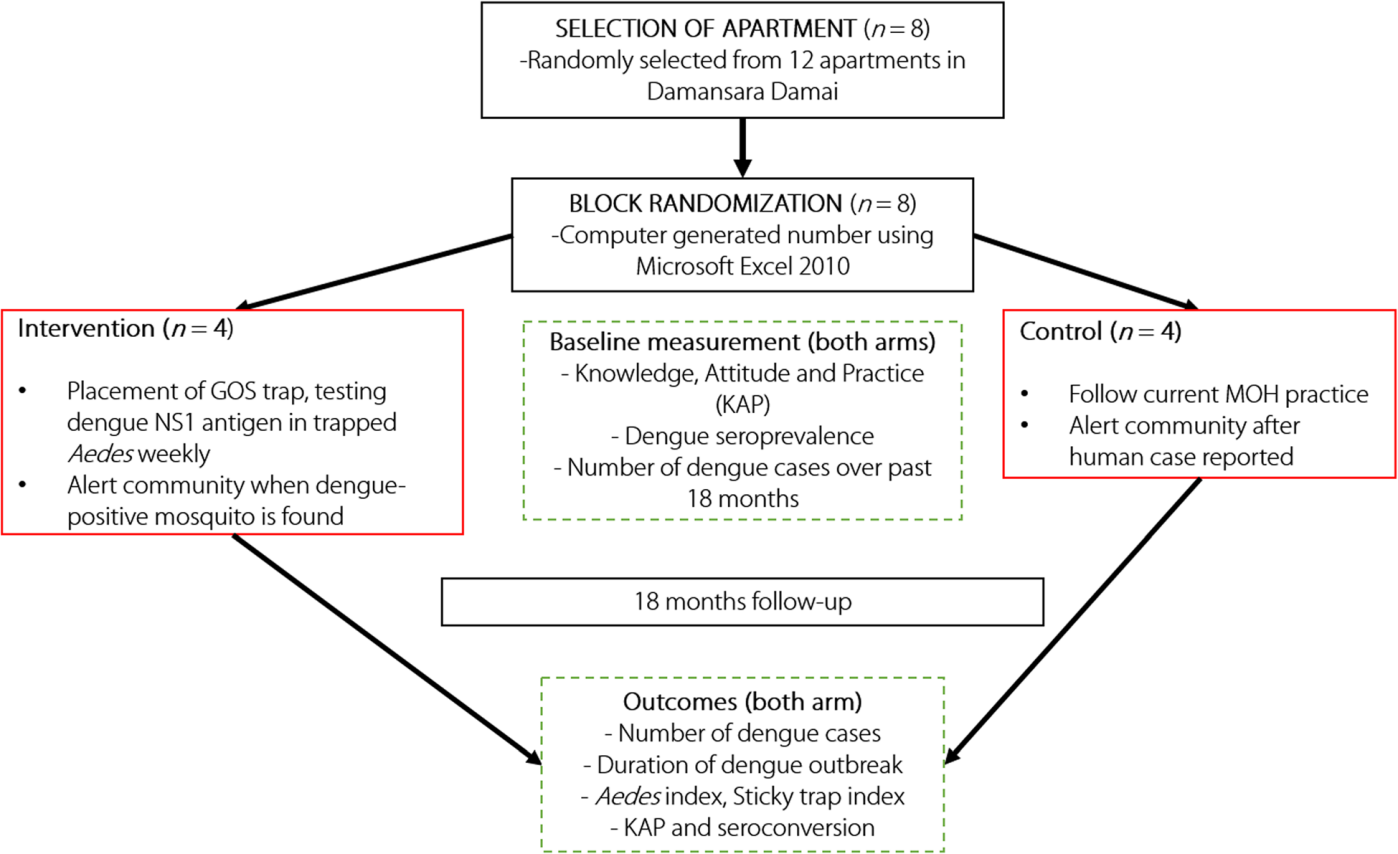
Flow chart of study procedure

The study procedure is demonstrated in Fig. 1.

7

### Study setting

**Fig. 2:**
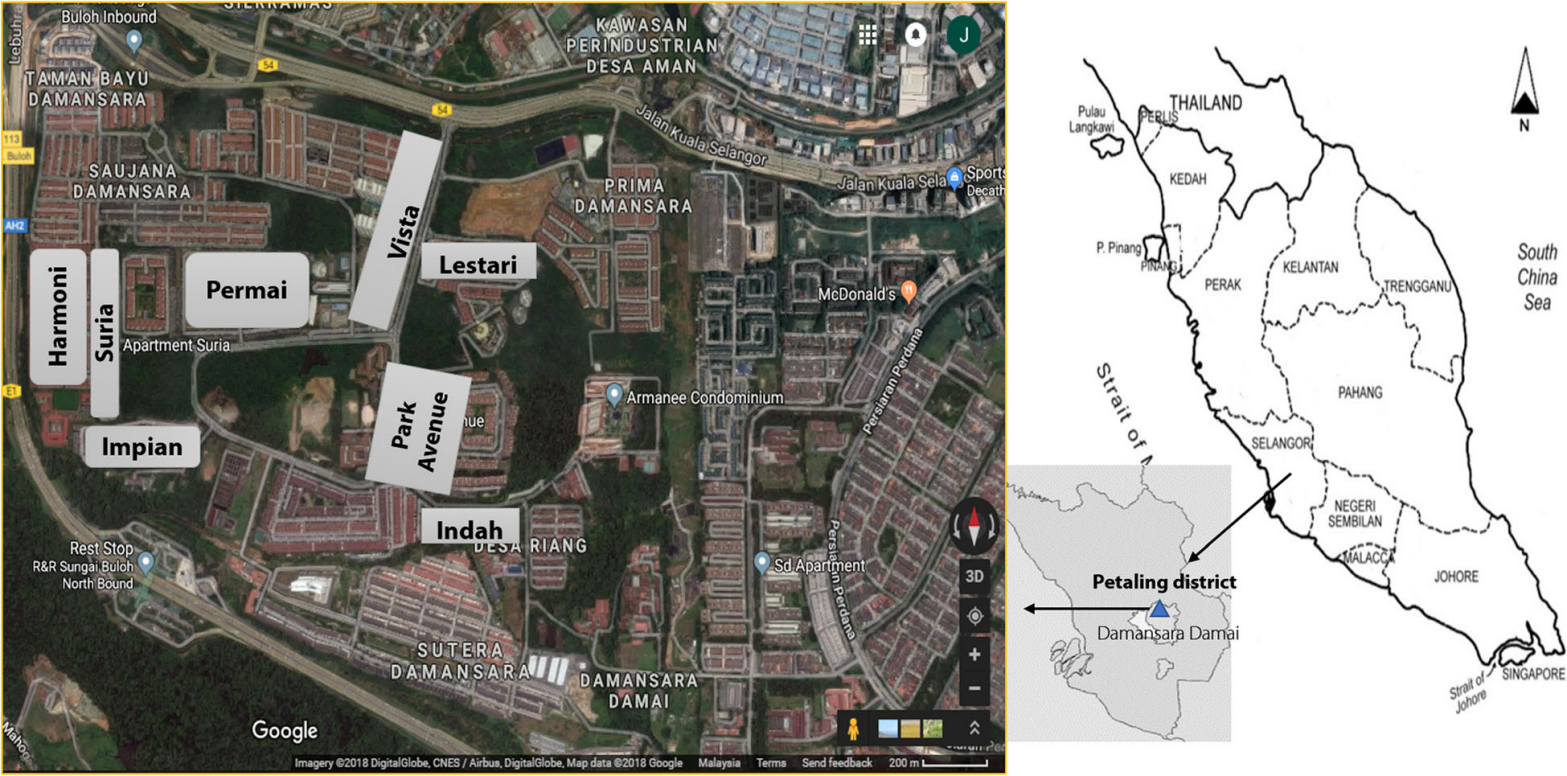
Map of study site. Insert: map of Selangor showing the study sites (apartments) in Damansara Damai, Petaling District

This trial is conducted at Damansara Damai in Petaling Jaya Utara 10 (3.1930°N, 101.5923°E), Petaling district, Selangor, Malaysia (Fig. 2). Of the nine districts, Petaling district is the major contributor to the dengue cases in Selangor [3]. Following discussions with the Petaling Jaya City Council, Damansara Damai was chosen in view of its “closed” area (there is only one main entrance and exit to this area), its status as a dengue hotspot and it having sufficient number of high-rised apartments.

In 2018, Damansara Damai has approximately 61 615 inhabitants in an area of 3.45 km^2^, translating into a population density of approximately 17 859 inhabitants/km^2^ (Development Planning Department, Petaling Jaya City Council, personal communication). There were 278 and 227 dengue cases reported respectively in 2018 and 2017 in this area (Vector Control Unit, Petaling Jaya City Council, personal comunication). Eight apartments were selected to be included in the cluster randomized controlled trial that is being carried out for 18 months.

### Sample size

The sample size was calculated based on that for a cluster randomized trial [31] using the Stata 13 statistical software (StataCorp, Texas, USA) [32]. The likely incidence and spatial variation of dengue were determined with reference to the number of confirmed cases in selected localities in Selangor from 2013 to 2017 (Vector Control Unit, Petaling Jaya City Council, personal communication). Specifically, the variations between localities in each year were utilized as a guide to estimate the extent of variations between trial clusters. Each cluster contained apartment blocks with similar population sizes. The minimum sample size required to detect a 60% reduction of dengue in the intervention arm with a power of 80% is 20 blocks per arm. From there, the sample size was inflated by 20%. Accordingly, a total of 48 blocks is required for the study.

For the interventional trial, it was estimated that on average, each housing unit will have 2% risk of dengue per year. To test the ability of the intervention to reduce the number of dengue cases, a sample size of 5710 units (2855 per arm) is required to achieve 80% power at an alpha of 0.05 (assuming a 50% risk reduction at the end of the intervention).

### Randomization

Initially, eight apartments with 7889 residential units in a total of 75 blocks were randomly selected and randomized into the intervention and control arms. Individual apartment was the item of randomization. The apartments were keyed into Microsoft Excel 2010 (Microsoft, Washington, USA), following which they were randomly allocated to the intervention and control arms using computer-generated numbers. Initially, Suria Apartment, Harmoni Apartment, Bayu Apartment, and Park Avenue Condominium were assigned to the intervention arm while Vista Apartment, Indah Apartment, Lestari Apartment, and Permai Apartment the control arm. In the event that an apartment declines to participate or drops out of the trial, an apartment which had similar numbers of cases per 1000 units and cases per block will be chosen as a replacement. The participants (residents/apartments) and the researchers are not blinded to the intervention.

### Recruitment

Following the receipt of ethical approval from the University Malaya Medical Center Medical Research Ethics Committee, meetings were conducted with the joint management body (JMB) of each apartment, during which written approvals were obtained for house-to-house visits as well as deployment of GOS traps. The JMB is in charge of the management and maintenance of an apartment. Concurrently, a complete schedule for the replacement of the GOS traps was given to the JMBs in an attempt to promote the involvement of the community in the study. Approvals from each JMBs were received at different time. Nonetheless, upon receipt of the first approval, the trial started in week 40 of year 2018. All selected apartments provided their consents except for Bayu Apartment. Consequently, Impian Apartment which had similar number of cases per 1000 units and cases per block, as well as similar apartment design as Bayu Apartment was chosen as the replacement. The details of the finalized apartments in both arms are presented in Table 1.

**Table 1:** Characteristics of the study arms and the number of traps in each apartment

	No. blocks	Total No. of units (residential units)	No. of floors in each block	No. of floors with traps in each block	No. of individual stairway leading to residential units	No. traps in each block	Total No. traps
Intervention							
Suria	8	767 (444)	5	2	6–8	12–16	114
Harmoni	19	2380 (2356)	5	2	NA	6	114
Park Avenue	2	316 (316)	17/18	6	NA	16–17	33
Impian	6	668(660)	5	2	NA	6	36
Total	**35**	**4131 (3776)**					
Control							
Vista	15	920 (736)	5	1–2	5–7	3–5	66
Lestari	9	2002 (1982)	5–15	2–6	NA	2–6	25
Indah	2	120 (120)	5	2	NA	2	4
Permai	12	1379 (1365)	5	2	NA	2–3	28
Total	**38**	**4421 (4203)**					

*No*. Number, *NA* Not applicable

### Eligibility criteria

All the residents/residential units of the eight sampled apartments are eligible for this study. Additionally, people who work at the study sites for at least 40 h per week are also eligible. They are approached via the JMB of each apartment, door-to-door visits or community-based events (like clean-ups and annual general meetings). All individuals who are 18 years old and above and able to provide informed consent are recruited. Conversely, those who are aged below 18 years and unable to provide informed consent are excluded.

### Intervention

Dengue control activities still take place as per the Ministry of Health’s vector control measures in both intervention and control arms throughout the trial. The measures may consist pyrethroid-fogging at the apartments where new dengue cases are reported. Larval surveys may also be conducted depending on the severity of the outbreaks and the availability of manpower [33].

#### Placement of GOS traps

A detailed description of the GOS trap is provided in reference [18] where it was initially tested as an *Aedes* surveillance tool for dengue control [18]. In this trial, the outer black container is a 700 ml black, round polypropylene plastic container (Diameter 110 mm × Height 87 mm). While the inner container is a 280 ml clear, round polypropylene plastic container (Diameter 110 mm × Height 46 mm). All traps are filled up till the bottom of the inner container with 10% hay infusion water made from 1-week-old hay infusion [34]. Each trap costs about Malaysian Ringgit (MYR) 1.50 (US dollar [USD] 0.375) and is reusable, except for the brown sticky paper which has to be replaced weekly.

Traps are placed where it is shaded and protected from rainfall, and ideally at locations where it is dark and away from human traffic. The GOS traps are deployed continuously and changed weekly for 18 months. The placement of traps and the number of traps deployed are shown in Table 1. The placement of traps varies according to the designs of the individual apartments. For the intervention arm, GOS traps are placed at the corridors of each block. Generally, for the apartments with common corridors, three traps are placed not more than 45 m apart at the corridors at every three floors (two at each end and one in the middle) [18], starting from the floor with residential units. As an example, a 5-storey block will have traps at two of its floors (Ground/Mezzanine and third floor), amounting to a total of six traps per block. There are a few exceptions: (i) If a block has five floors, and residential units are not found starting from the ground floor but the first floor, traps will be set on this floor and on the 3rd floor; (ii) For the shop apartments (Suria), two traps will be placed on every stairway (Mezzanine/1st floor and 2nd/3rd floor) leading to the residential units; (iii) For floors which are short i.e. corridor is less than 25 m in length (in Park Avenue), only two traps are set on the said floors. A manpower of four is enough to cover 19 blocks of an apartment within one hour.

#### Detection of dengue non-structural 1 (NS1) antigen in pooled Aedes mosquitoes trapped by the GOS traps

During collection of the GOS traps, they are examined for trapped *Aedes* and *Culex* mosquitoes. The numbers of missing sticky papers of the traps in each block will be recorded. The *Aedes* mosquitoes will be identified to species level, and the numbers of each mosquito species of interest in each trap will be recorded. However, only female *Ae. aegypti* and *Ae. albopictus* mosquitoes will be processed as described below.

The abdomens of the *Aedes* mosquitoes will be separated from the thoraxes and pooled. Each head-thorax will be kept in individual 1.5 ml tubes and stored at − 20 °C. The pooled abdomens will be homogenized and tested for the presence of dengue NS1 antigen using the SD Biosensor Standard Q Dengue NS1 test kit (Gyeonggi-Do, South Korea). When an abdomen pool is positive, the same test will be repeated for each corresponding head-thorax in an attempt to identify the specific/proximal location (trap) where the dengue NS1-positive mosquito was found.

Depending on the number of positive traps and number of mosquitoes trapped, the above can be completed within four hours by four personnel. Overall, setting traps and collection of traps in 19 blocks of an apartment; identification and dissection of mosquitoes; and the detection of dengue NS1 antigen in pooled mosquitoes require approximately 20 man-hours.

#### Notifications of the presence of dengue-positive Aedes

When the test above is positive, flyers and posters will be disseminated within two days to notify the affected apartment’s management and residents that dengue-positive mosquito (es) is detected. The Vector Control Unit of the Petaling Jaya City Council will also be informed. Consequently, pyrethroid fogging may be carried out by them. The flyers/posters will contain information of (i) the exact block(s) where the dengue-positive mosquito (es) was found, as well as (ii) precautionary measures for residents to protect themselves from mosquito bites. The posters will be put up at strategic locations such as the notice boards of each block. The management will also make use of social media such as Whatsapp or Facebook to disseminate the said information to the residents. Concurrently, mosquito repellants [Fumakilla Nobite Lotion, active ingredient: 10% icaridin (Fumakilla Malaysia Berhad, Penang, Malaysia); and NATMOS anti mosquito spray, active ingredient: lemongrass and lemon eucalyptus oil (OPC Resources Sdn Bhd, Penang, Malaysia)] and flyers will be distributed door-to-door. The residents will be educated and reminded to take precautionary measures against dengue as well as perform mosquito search-and-destroy activities.

#### Control arm

In the control arm, a small number of GOS traps are deployed in each block, for entomological survey only. The number of GOS traps placed per block corresponds to one-third of that in the intervention arm. The traps are only deployed and left for a week once every month at the same location. The traps are placed randomly on floors as per the intervention arm (Table 1). Thus, a single floor/stairway may have zero to two traps. As an example, a five-storey block in the control arm will have two traps (which corresponds to one-third of the six traps to be placed if the block is in the intervention arm) set randomly at either one (Ground/first/Mezzanine or third floor) or two floors (Ground/first/Mezzanine and third floor) of the block.

### Outcomes

The primary outcome of the trial will be measured in terms of the percentage change in the (i) number of dengue cases and (ii) durations of dengue outbreaks between the control and intervention arms. Data will be collected weekly through study completion for 18 months and analysis will be performed thereafter.

The secondary outcome concerns the change in (i) adult sticky trap index (ASTI; number of traps with adult *Aedes* mosquitoes in total, *Ae. aegypti* only and *Ae. albopictus* only every week in each apartment and collectively in the intervention arm); (ii) adult index (AI; number of female adult *Aedes* mosquito in total, *Ae. aegypti* only and *Ae. albopictus* only collected every week using GOS trap in each apartment and collectively in the intervention arm); and (iii) dengue-positive trap index (number of traps with *Aedes* mosquito positive for dengue NS1 per apartment collected every week). The numbers of *Ae. aegypti* and *Ae. albopictus* will be recorded weekly based on the source traps and apartments for 18 months until the completion of the study, when analysis will be done. Subsequently, the changes in the AI and ASTI throughout the study will be calculated.

Other outcomes which will be obtained is change in levels of dengue-related knowledge, attitudes and practices (KAP) of the residents using a self-administered questionnaire. Levels of KAP will be expressed as percentage of correct answers for each domain. Individuals whose scores for each domain exceed 80% will be considered having good KAP. The level of KAP of residents in the intervention and control arms will be determined six months after recruitment. Beginning of the 16th month of the trial (January 2020), a new set of questionnaire will be administered to the residents to gauge their levels of KAP again and their receptiveness towards the new dengue surveillance method. The levels of KAP before and after will be compared 3 months after recruitment for post-trial survey.

### Data collection

#### Adult Aedes surveillance using the GOS traps

As mentioned above, all traps will be replaced weekly. The GOS traps and trapped mosquitoes will be processed in the lab. Mosquitoes caught by these traps will be identified by trained researchers. The number of missing traps (missing brown sticky paper) will be recorded for each block. Only the numbers of *Ae. aegypti*, *Ae. albopictus* and *Culex* spp. mosquitoes in each trap will be recorded whereas only the female *Aedes* mosquitoes will be processed and stored. During dissection of the mosquito under the stereomicroscope, the species and number of mosquitoes will be checked again, making sure the records tally and none was missed during the first round of inspection. Then, the abdomens of the *Aedes* mosquitoes will be separated from the thoraxes and up to 5–7 abdomens will be pooled according to species (i.e. one pool of *Ae. aegypti*, another pool of *Ae. albopictus*). The thorax and head will be stored in individual tubes. The pooled abdomens will be homogenized in 150 μl of phosphate-buffered saline (PBS) using a pellet pestle. The homogenate will be centrifuged at 8000 rpm for 1 min at room temperature. One hundred microlitres of the supernatant will be used on a SD Biosensor Standard Q Dengue NS1 test kit to detect dengue NS1 antigen in the pool of abdomens. The presence of a “Test” band — however faint — along with a “Control” band, will be regarded as positive. Reading is taken after 15–20 min, after which the result is considered invalid. At least two persons will view the result. In the event that there is discordance, a third individual will be sought and the result will be determind by this person. When the abdomen pool is positive, the head and thorax corresponding to the mosquito abdomens in that pool, will be homogenized individually in 120 μl of PBS, centrifuged at 8000 rpm for 1 min and tested for dengue NS1 antigen as above. This allows us to identify the GOS trap(s) from which the positive mosquito (es) originated. If a dengue NS1-positive mosquito is found in the intervention arm, the apartment in question will be notified of the result. Steps will be taken, as detailed in the **Intervention** section.

#### Dengue virus detection and serotyping

The dengue NS1-positive mosquito abdomen pools will be further analysed using PCR. Viral RNA will be extracted from the remaining homogenate using TRIzol, following the manufacturer’s protocol. Five μl of RNA will be used for the first RT-PCR reaction, followed by a nested PCR using the primers stated by Klungthong et al. (2015) [35] for serotyping the dengue virus.

#### Dengue cases in the study sites

The weekly number of notified dengue cases and duration of dengue outbreaks in the study sites will be obtained from the District Health office. Dengue fever is a notifiable disease in Malaysia, and all suspected/confirmed dengue fever cases have to be notified. Confirmed dengue is defined as a case compatible with clinical description of dengue and laboratory-confirmed with any of the following: detection of dengue NS1, dengue IgM/IgG seroconversion in paired sera, detection of dengue IgM and IgG in a single sample and PCR, among others [36]. The data obtained will be used for analysis to determine if the intervention is able to reduce the number and duration of dengue outbreaks.

#### Knowledge, attitude and practice (KAP) on dengue and dengue seroprevalence of the residents in the study sites

Assuming that the baseline knowledge of dengue in the sample population was 50%, the minimum sample size required to attain 80% power at an alpha of 0.05 is 384. This value was increased to 460 following a 20% inflation. With reference to the dengue seroprevalence study, it was assumed that the baseline seroprevalence of dengue in the sample population was 60%. Ergo, the minimum sample size needed to achieve the aforementioned power and alpha is 369. Following a 20% inflation, the value was increased to 443. Therefore, the overall minimum number of residents needed in this part of the research is 460.

In the initial six months of the interventional trial, KAP questionnaires had been given to the residents at every apartment (both intervention and control arms) via door-to-door visits. Recruitment was also performed during community-based events at the apartments (e.g. health screenings, clean-ups, annual general meetings, etc.). Following receipt of informed consent from individual residents, a self-administered KAP questionnaire was provided for them to answer on the spot. The questionnaire used had been adapted from that of Zaki et al. (2019) [37]. This questionnaire had been pilot tested in a community similar to the current study population. All sections had Cronbach’s alpha values of more than 0.60 and are considered acceptable. Test re-test reliability of the questionnaire was performed among a sample of 50 participants with intraclass correlation coefficient (ICC) ranging from 0.6 to 1.0, showing that all items achieved moderate to excellent reliability. The questionnaire has three sections. The first section with eight questions collects personal information such as gender, age, race, education level, income, history of dengue fever and others. The second section gauges the respondent’s knowledge on dengue with 28 dichotomous or single-best-answer questions. The last section assesses attitude and practice of dengue prevention with 16 questions. Upon receipt of the completed questionnaires, a code was assigned to each. The information was keyed and stored in cloud storage, whereby only the authors have access to. The data will be analyzed using IBM SPSS Statistics software version 23 (IBM Corporation, New York, USA).

The participants were free to choose to complete the questionnaire survey only, or undergo venepuncture only for dengue seroprevalence, or participate in both. About 3 ml of venous blood from each consented individual was taken in a plain blood tube for dengue seroprevalence by a trained personnel. The blood sample was coded and tallied with that of the questionnaire (if respondent participate in both activities). The blood tubes were centrifuged at 4000 rpm for 4 min, and the sera were aliquoted and stored at ≤ − 70 °C, until use in the dengue IgG and IgM ELISA tests. If the sera were not processed on the same day as blood-taking, the blood tubes were stored at 4 °C and processed the next day. ELISA will be performed according to the manufacturer’s protocol in technical duplicates. Results of the ELISA test will be informed to the respective participants via a phone call, text message or verbally in a visit to their premises.

#### Post-interventional survey and measuring dengue seroconversions

At the end of the intervention (at the beginning of the 16th month of trial), a self-administered KAP questionnaire will be given to the residents of all apartments again. For participants living in the intervention arm, additional questions will be asked to obtain their feedback on this new method of dengue surveillance. The participants will also be asked if they are willing to provide blood to investigate dengue seroconversion. Blood will be taken, processed and analysed similarly as stated above for the study on dengue seroprevalence. Recruitment and analysis will also be performed as above.

### Data management

Each GOS trap is given an identification number (indicating the apartment and house unit number close by where it is placed). As stated above, each respondent for the KAP survey and/or dengue seroprevalence was also given a unique Identification (ID) number and the samples/data collected from them were labelled with the corresponding ID numbers. Data from KAP questionnaires, GOS trap collections and blood samplings is collected on paper forms, which is subsequently keyed and stored in cloud storage, whereby only the authors have access. All hardcopy data is placed in a locked room. Procedures for data management can be sourced from the corresponding author.

### Statistical analyses

The effectiveness of the intervention will be determined using two outcome measures, namely percentage change in number of dengue cases and percentage change in the duration of dengue outbreaks in the intervention arm during the study period as compared to the previous years; and also compared to that of control arm. The number of dengue cases will be obtained from the Health District Office, where these cases are notified to them. Both measures will be described in terms of means ± standard deviations (or medians and interquartile ranges). Unpaired *t*-tests will be used to determine the presence of significant differences in the means (or medians). Additionally, the rate of disease and the rate ratio will also be computed to indicate any difference in the outcome in both arms, followed by calculation of the protective efficacy of the intervention. [38]

For secondary outcomes, the difference and change in adult *Aedes* density in the intervention arm will be assesed using the ASTI, AI and dengue-positive trap indices. The numbers of *Ae. aegypti* and *Ae. albopictus* will be recorded weekly based on the source traps and apartments. All these will be analyzed at the arm and apartment level using weekly data. Additionally, the indices and cases per week in each apartment will be analysed by generalised linear mixed model (GLMM) as in [27].

The dengue-related KAP of the residents will be expressed as percentages of correct answers for each of the domains. Subsequently, these will be presented in terms of means ± standard deviations. For the intervention arm, paired *t*-test will be performed to determine the presence of significant improvement in the mean scores of both domains with regards to the post-interventional survey. On another note, unpaired *t*-tests will be performed between (1) the pre-intervention test scores of the intervention arm and that of the control arm, as well as (2) the post-intervention test scores of the intervention arm and the scores of the control arm to detect the presence of significant differences between them. Individuals whose scores for each domain exceed 80% will be considered to have good knowledge and/or attitudes/practises accordingly. Subsequently, the percentages of residents in both arms who have good knowledge and attitudes/practises will be determined. From there, chi-squared tests will be performed to determine the odds ratios (with 95% confidence intervals) of the occurrence of good knowledge and attitudes/practises in the intervention arm with reference to those of the control arm. Association between KAP with socio-demographic variables and past infection (from dengue seroprevalence data) will also be determined by calculating the odds ratios and the respective 95% confidence intervals by logistic regression. These analyses will be performed using IBM SPSS Statistics software version 23.

To gauge the receptiveness of the community to this new intervention, the percentage of respondents whose answers indicate support will be calculated. Chi-squared tests and/or logistic regression will also be performed to determine the odds ratios (with 95% confidence intervals) to determine any association between receptiveness with socio-demographic variables.

### Monitoring, harms and auditing

Data monitoring will be conducted by the research team that is directly involved in the progress of the trial; drafting and carrying out plans; and solving ethical and unforeseen events. The researchers will meet monthly to discuss progress and any issue that arises. There is no interim analyses and stopping guidelines.

This trial poses minimal risk to the participants. The GOS trap is made of safe material, none which will cause major harm to any individual. The sticky insect glue is non-toxic and can be removed from hands and tools using vegetable oil, followed by soap. Hence, there is no obvious forseeable harm from the intervention. Only minor discomfort from the venepuncture for dengue serology is anticipated, which will not impact the routine physical and psychological performance of the participants. However, if there should be any adverse event or complaint, contacts of the investigators have been given out to the residents in the participant information sheet and also through correspondence with the JMBs. In view of these, no criteria has been set for terminating or modifying the interventions, nor is there any endpoint guidelines.

There will be no auditing for this trial.

## Discussion

Adult mosquito traps have been deployed and tested in various settings. These traps come in different designs. In a randomized control trial using BG-Sentinel traps, their deployment has slightly reduced the density of *Aedes* in the experimental area as compared to its control counterpart [19]. Other autocidal traps such as the In2Care mosquito trap [39] and Gravid *Aedes* Trap [40] which contains insecticides, larvicides and/or biological control agents that kill the adults and larvae, have also been found to be useful for vector surveillance/control. Similarly, sticky traps were reported to be as effective as [41] or even more sensitive than standard ovitraps in detecting *Ae. aegypti* [42]. Variants of the sticky trap, such as *Aedes*TraP [43] and MosquiTRAP [21] were more sensitive than larval survey at detecting presence of *Aedes*, besides demonstrating superiority in predicting dengue transmission risks [44]. When comparing efficiency of these sticky traps, MosquiTRAP captured a higher number of female *Ae. aegypti* per trap than Adultrap, although both were considered efficient and reliable [45]. In a separate study, all four investigated traps (Adultrap, BG-Sentinel, MosquiTRAP and Ovitrap) rarely produced null indices and performed better than house index in estimating seasonal abundance of mosquito [46]. Therefore, these adult traps are considered valuable tools and the choice of trap depends on specificity, low cost, ease of use, and consistency [6].

However, some of these traps are expensive and may not be cost-effective for a surveillance programme. Others could be relatively labor-intensive, dependent on handler (backpack aspirators), while some uses electricity (BG-Sentinel). Being patent-free and insecticide/larvicide-free, the GOS trap is a cheap and simple trap to make and deploy, making it more amicable to the public. In addition, a physical barrier is present to prevent emergence of adult mosquitoes if eggs are laid. Although, double sticky traps collected significantly more adults than standard sticky trap (model similar to GOS trap) in Trinidad, West Indies [28], this model was evaluated in Malaysia, and was found to be inferior compared to other models and the standard sticky trap [47]. It was found that a small, simple ovitrap with the sticky paper was most attractive to the *Aedes*. However, it was felt that it is not practical to use such trap as it can allow larvae to develop and adult mosquitoes to escape. Thus, the current design of the GOS trap, which is slightly smaller than the earlier version [27] is adopted, similar to that currently being used for management of dengue clusters in Singapore [22].

Nonetheless, GOS trap, as with other adult traps, do not provide absolute measurement of mosquito abundance as they compete with other natural or cryptic breeding sites. Therefore, this trap is black in colour and also hay infusion water is used. It has been shown that these factors make the traps more attractive to *Aedes* mosquitoes [48, 49], thereby increasing the probability of the mosquitoes choosing the GOS trap over other containers. Besides, trap indices rather than mosquito counts will be the focus for analyses. Still, these traps that capture adults can obtain estimates of population directly involved in dengue transmission. And because gravid mosquitoes are trapped, these mosquitoes which have taken a previous bloodmeal (possibly an infectious one), allow us to survey dengue transmission in the area. Recent works also encourage using arbovirus prevalence in mosquitoes as a proxy for human infections [50–52].

Hence, we anticipate the GOS traps to be able to bring down dengue cases, by trapping (infected) gravid *Aedes* mosquitoes. Although some studies collectively indicate only a 22% positive correlation between vector indices and dengue cases [53], other studies found significant correlation between weekly *Aedes* abundance (in egg counts and ovitrap index) with number of confirmed dengue cases [53, 54]. Increase in *Ae. aegypti* adult catches, were observed along with increased number of dengue cases [53] and adult *Ae. albopictus* abundance was also positively correlated with dengue fever incidence, in Zhejiang, China [55]. In addition, average adult mosquito abundance is consistently higher in a dengue hotspot compared to a neighbouring low-transmission spot, in Taiwan, China [56]. Similarly, mosquito densities and rates of chikungunya virus detection were significantly higher in communities without vector control than in treated communities (using autocidal gravid trap) [50, 51]. A research which employed the Centers for Disease Control (CDC) autocidal gravid trap has also reported a significant reduction in the number of dengue cases (by 53–70%) in the interventional area vis-à-vis the control area [25].

However, it should be noted that, the GOS trap is not the only intervention of this trial. The intervention includes early public alert of the communities and health authorities, to carry out preventive and protective measures. This may promote the participation of the community and encourage them to be more responsible in the surveillance of dengue vectors. Knowledge and awareness are integral parts of a vector control program. Inadequate knowledge and poor practice contribute to the spread of dengue fever [57]. Therefore, engaging the community and educating the public in vector surveillance/control should be done to ensure in-built sustainability and active health promotion. Additionally, the GOS traps are very cheap and can be handled by the community with help from the health authorities. In the future, if this method of surveillance is to be established, dengue NS1 antigen detection can be performed directly on the pooled mosquitoes without dissection. Because of its simplicity, the detection of dengue antigen in the mosquitoes can also be carried out by the community. Thus, making it an integrated vector management system and not a top-down approach.

The study sites are multistorey dwellings where population per unit area is likely to be higher, thus surveillance should be different than landed properties [58]. In many urban areas high rise dwellings are becoming popular and it will be tedious for the authorities to carry out larval surveys as was done decades ago. Moreover, the *Aedes* mosquitoes now have cryptic breeding sites [59] which makes it even more difficult to adopt the search and destroy strategy. In this case, surveillance using the GOS traps should be able to efficiently provide sufficient coverage. Furthermore, trapping dengue-infected mosquitoes using the GOS trap, helps to halt the dengue transmission carried by the mosquito, an added benefit of using a capture-kill trap.

It is envisaged that the results of this randomized controlled trial will provide a new proactive, cheap and targeted surveillance tool for the prevention and control of dengue outbreaks. It is an added advantage because it will not only control the spread of dengue but also Zika and chikungunya viruses. However, some problems have been encountered, which include, missing traps, tampering of traps, unwillingness of residents to have traps placed directly beside their premises etc. We managed to circumvent some of these issues by spending time to explain and interact with the residents. Some of the traps were also moved to an adjacent unit or 2–3 m away.

Although the *Aedes* house index is now much lower than it was decades ago [35], the number of cases are on the increase. One plausible reason may be due to the fact that current cases are occurring in high rise apartments where people are living close to each other and thus small number of mosquitoes are sufficient to keep the transmission circulating. It is thus envisaged that the current trial will be able to provide insight and solutions to reduce the number of dengue outbreaks.

## Additional file

Additional file 1: Multilingual abstracts in the five official working languages of the United Nations. (PDF 346 kb)

## Data Availability

Not applicable.

## Abbreviations

AI: Adult index
ASTI: Adult sticky trap index
GOS: Gravid oviposition sticky
KAP: Knowledge, attitude, practise
NS1: Dengue non-structural 1 antigen
