# Supplementary material for: Gravid oviposition sticky trap and dengue non-structural 1 antigen test for early surveillance of dengue in multi-storey dwellings: study protocol of a cluster randomized controlled trial
Source: Infect Dis Poverty. 2019 Sep 3;8:71. doi: 10.1186/s40249-019-0584-y (PMC6720065; doi:10.1186/s40249-019-0584-y)
Supplement: Supplementary file 1 — Multilingual abstracts in the five official working languages of the United Nations. (PDF 346 kb) [file 40249_2019_584_MOESM1_ESM.pdf]

Translation of the abstract into the five official working languages of the United Nations

مصيدة **Gravid Oviposition** اللزجة واختبار المواد المضادة لفيروس الضنك **NS1** لمراقبة مبكرة لفيروس الضنك في الاماكن المتعددة: دراسة المشاهد الأولية ل للتجربة المدروسة المُختارة عشوائيا

جوناثان وي كينت ليو ، سيفانسواري سيلفاراجو ، وينج تان ، رافدة أحمد زكي وإندرا فيثيلينجام

الخلاصة

الخلفية: تعد حمى الضنك من الأمراض العالمية ، التى تنقل بواسطةبعوضةالزاعجة . فى عام 2018 رصدت 80615 حالة حمى ضنك فى ماليزيا أدت الى 147 حالة وفاة] تعتمد برامج المراقبة القومية على استقصائات يرقة البعوضة والاخطارات المؤكدة للعدوى البشرية معمليا. كما ان البرامج التفاعليه الموجودة تفتقر الى الحساسية والتنسيق المُسبق. هناك حاجة لطرق أكثر فاعلية لمراقبة حاملوا حمى الضنك و السيطرة عليها

اساليب العلاج :تدبر تجربة تداخلية متوازية ، عشوائية عنقودية لمدة 18 شهر دامانسارا داماي ، سيلانغور ، ماليزيا لتحديد فعالية استخدام مصيدة gravid oviposition اللزجة واختبار المواد المضادة لحمى الضنك غير المهيكلة لمراقبة مبكرة لحمى الضنك بين بعوضة الزاعجة لتقليل حالات التفشي الخاصة بحمى الضنك خُصصت ثمانية اماكن إقامية بشكل عشوائي للتداخل والحد من الضرر. وُضعت فخاخ GOS في الشقق لجمع البعوض اسبوعيا ، متابعة أي من مضادات فيروس الضنك وُجدت في تلك البعوض. فى حالة اكتشاف بعوض حامل لحمى الضنك يتم ابلاغ المجتمع للقيام بعمليات وقائية للبحث و القضاء على ناقلى المرض تتعلق النتيجة الأولية بنسبة تغير حالات فيروس الضنك و مده نتيجة الفيروس في حين تتضمن مقاييس النتائج الأخري التغير في كثافة عتبة البعوضة والتغيرات في الفيروس المتعارف عليها، السلوك والممارسة بين سكان العنقود.

مناقشة: هذه مراقبة مسبقة ومبكره لفيروس الضنك في ناقلات المرض بالبعوضة التي لم تعتمد علي إخطارات الحالات الخاصة بفيروس الضن.. يجب ان تكون المراقبة بإستخدام مصائد GOS قادرة على توفير تغطية كافية للمسكان متعددة الطوابق حيث من الممكن ان السكان في المنطقة الواحدة ان يتضاعفوا. لاوة على ذلك، تُساعد محاصرة البعوض المصاب بواسطه مصائد GOS على وقف انتقال فيروس الضنك بواسطة البعوض. من المتصور أن نتائج التجربة المحكومة عشوائيا ستوفر إستباقية جديدة ، اداه رخيصة ومحددة لمنع العدوى والحد من نتائج فيروس الضنك

Translated from English version into Arabic by Abdallah Hamdy, Revised by Ahmed Ibrahim, through

TRANSLATORS WITHOUT BORDERS

孕蚊产卵粘滞器和登革 NS1 抗原检测用于多层住宅的登革热早期监测:一组随机对照试验研究方案

Jonathan Wee Kent Liew, Sivaneswari Selvarajoo, Wing Tan, Rafdzah Ahmad Zaki and Indra Vythilingam

摘要

引言：登革热是一种由媒介伊蚊传播的疾病，呈全球分布。2018 年，马来西亚共确诊 80615 例登革热病例，死亡 147 例。目前，全国范围的监测项目仍依赖于伊蚊幼虫调查和经实验室

确诊的病例感染。然而。目前现有的应对措施似乎缺乏敏感性和主动性。因此需要更为有效的登革热病媒监测或控制方法。
方法：在马来西亚雪兰莪州达曼萨拉达迈（Damansara Damai, Selangor），正在进行一项为期 18 个月的平行、集群、随机对照干预试验，用以确定使用孕蚊产卵黏性(GOS)诱捕器和登革热非结构性 1 抗原(NS1)检测对伊蚊登革热早期监测的有效性，以期减少登革热暴发。在这项实验中，将 8 套住宅公寓随机分配到干预和控制组，在公寓内设置 GOS 陷阱，每周收集伊蚊，然后在这些蚊子体内检测到登革热 NS1 抗原。当发现呈登革热阳性的伊蚊时，当局会建议市民采取媒介搜寻及灭蚊和防护措施。主要结果涉及(i)登革热病例数和(ii)登革热暴发持续时间的百分比变化。其它测量结果包括伊蚊密度阈值的变化，以及集群居民中与登革热相关的知识、态度和行为的变化。
结论：这种主动和早期登革热媒介监测不依赖登革热病例通报。使用 GOS 陷阱进行的监测能有效地为单位面积人口较多的多层住宅提供足够的覆盖。此外，利用 GOS 诱捕器诱捕感染登革病毒的伊蚊有助于阻断登革热的传播。这一随机对照试验的结果将有望为预防和控制登革热暴发提供一种新的主动、廉价且有针对性的监测工具。

Translated from English version into Chinese by Xin-Yu Feng, edited by Jin Chen

CHINESE CENTER FOR DISEASE CONTROL AND PREVENTION
NATIONAL INSTITUTE OF PARASITIC DISEASES

Test du piège collant Gravid Oviposition et de l'antigène NS1 de la dengue pour la surveillance précoce de la dengue dans les habitations à plusieurs étages: protocole d'étude d'un essai contrôlé randomisé par grappes

Jonathan Wee Kent Liew, Sivaneswari Selvarajoo, Wing Tan, Rafdzah Ahmad Zaki et Indra Vythilingam

RÉSUMÉ

Contexte: La dengue est une maladie mondiale, transmise par les vecteurs *Aedes*. En 2018, il y avait 80 615 cas de dengue et 147 décès en Malaisie. À l’heure actuelle, les programmes de surveillance à l'échelle nationale dépendent des enquêtes sur les larves *d'Aedes* et des notifications d'infections humaines confirmées en laboratoire. Les programmes réactifs existants semblent manquer de sensibilité et de proactivité. Des méthodes plus efficaces de surveillance et de lutte contre les vecteurs de la dengue sont nécessaires.
Méthodes : Un essai d'intervention parallèle, en grappes, contrôlé et randomisé, est en cours pendant 18 mois à Damansara Damai, Selangor, Malaisie,, afin de déterminer l’efficacité de l’utilisation du piège collant à oviposition gravide (GOS) et du test d’antigène non structural 1 (NS1) de la dengue dans la surveillance précoce de la dengue chez les moustiques Aedes afin de réduire les épidémies de dengue.Huit appartements résidentiels ont été assignés au hasard aux groupes d’intervention et de contrôle. Des pièges GOS sont installés dans les appartements pour recueillir l’*Aedes* chaque semaine, après quoi l’antigène NS1 de la dengue est détecté chez ces moustiques. Lorsqu'un moustique atteint de la dengue est détecté, on conseillera à la communauté d'effectuer des recherches et de détruire les vecteurs et de prendre des mesures de protection. Le principal résultat concerne la variation en pourcentage du (i) nombre de cas de dengue et (ii) de la durée des foyers de dengue.

Alors que d'autres mesures des résultats incluent le changement du seuil de densité de l'Aedes et les changements dans les connaissances, les attitudes et les pratiques liées à la dengue parmi les habitants de la grappe.
Discussion : Il s'agit d'une surveillance proactive et précoce de la dengue chez le moustique vecteur qui ne repose pas sur la notification des cas de dengue. La surveillance à l'aide des pièges du GOS devrait être en mesure d'assurer efficacement une couverture suffisante pour les habitations à plusieurs étages où la population par unité de surface est susceptible d'être plus élevée. De plus, le piégeage des moustiques infectés par la dengue à l'aide du piège GOS permet d'arrêter la transmission de la dengue véhiculée par le moustique. Les résultats de cet essai contrôlé randomisé devraient fournir un nouvel outil de surveillance proactif, bon marché et ciblé pour la prévention et le contrôle des épidémies de dengue.

Translated from English version into French by Suzanne Assenat, Revised by Eric Ragu, through

Клейкая ловушка для откладывания яиц и тест на антиген NS1 вируса денге для раннего наблюдения за случаями заражения лихорадкой денге в многоэтажных домах: протокол исследования кластерного рандомизированного контролируемого исследования

Jonathan Wee Kent Liew, Sivaneswari Selvarajoo, Wing Tan, Rafdzah Ahmad Zaki and Indra Vythilingam

АННОТАЦИЯ

Справочная информация: Лихорадка денге представляет собой глобальное заболевание, переносчиком которого является комар вида *Aedes* . В 2018 г. было 80 615 случаев заражения лихорадкой денге в Малайзии, 147 из них — со смертельным исходом. На данный момент общенациональные программы эпиднадзора зависят от обследований личинок комаров вида *Aedes* и сообщений о лабораторно подтвержденных случаях заражения людей. Существующие реактивные программы оказались недостаточно чувствительными и проактивными. Необходимы более эффективные методы наблюдения/контроля за переносчиком лихорадки денге.
Методы. В г. Дамансара-Дамай, в штате Селангор, в Малайзии, в течение 18 месяцев проводилось параллельное кластерное рандомизированное контролируемое интервенционное исследование для определения эффективности использования клейкой ловушки для откладывания яиц и теста на неструктурный 1 (NS1) антиген вируса денге для его раннего выявления у комаров вида *Aedes* и снижения вспышек лихорадки денге. Восемь жилых домов случайным образом распределили в экспериментальную и контрольную группы. Ловушки для откладывания яиц были установлены в домах для еженедельного сбора яиц *Aedes* , после чего у этих комаров определяли наличие NS1 антигена денге. При

обнаружении положительного по денге комара, в сообществе рекомендуется применить меры по поиску и уничтожению переносчиков, а также защитные мероприятия. Основной результат заключается в возможности изменения процентного значения 1) количества случаев заражения денге и 2) продолжительности вспышек лихорадки денге. При этом иные результаты включали изменение в пороге плотности популяций комаров вида *Aedes* и изменения в знаниях о лихорадке денге, отношении и практиках среди жителей кластеров.

Обсуждение: Это проактивное и раннее выявление денге у комаров-переносчиков, которое не основывается на сообщениях о случаях заражения лихорадкой денге. Наблюдение с помощью ловушек для откладывания яиц должно быть в состоянии эффективно обеспечить достаточный охват многоэтажных жилищ, где численность населения на единицу площади, вероятно, будет выше. Более того, улавливание комаров-переносчиков, инфицированных денге, с помощью ловушки для откладывания яиц помогает остановить передачу лихорадки денге этими комарами. Предполагается, что благодаря результатам данного рандомизированного контролируемого испытания будет предоставлено новое проактивное, дешевое и целевое средство эпиднадзора для предотвращения вспышек денге и борьбы с ними.

Translated from English version into Russian by Veronika Demeshchyk, Revised by Anna Kukharchuk, through

TRANSLATORS WITHOUT BORDERS

Las ovitrampas adhesivas para hembras grávidas y la prueba del antígeno NS1 del dengue para la vigilancia precoz del dengue en viviendas de varias plantas: protocolo de estudio de un ensayo controlado aleatorio por grupos

Jonathan Wee Kent Liew, Sivaneswari Selvarajoo, Wing Tan, Rafdzah Ahmad Zaki e Indra Vythilingam

RESUMEN

Antecedentes: el dengue es una enfermedad mundial, transmitida por los vectores *Aedes*. En 2018, hubo 80 615 casos de dengue en Malasia, 147 de ellos fallecieron. Actualmente, los programas de vigilancia nacional dependen de los estudios sobre larvas *Aedes* y los avisos de infecciones humanas confirmadas en laboratorio. Los programas reactivos existentes parecen carecer de sensibilidad y proactividad. Se necesitan métodos de vigilancia / control de vectores del dengue más eficaces.

Métodos: durante 18 meses se ha realizado un estudio intervencional controlado aleatorio por grupos paralelo en Damansara Damai, Selangor (Malasia), para determinar la eficacia de utilizar ovitrampas adhesivas y la prueba del antígeno no estructural 1 (NS1) del dengue para la vigilancia precoz del dengue en los mosquitos *Aedes* para reducir los brotes de dengue. Se asignaron aleatoriamente ocho residencias a los grupos de control y de intervención. Las ovitrampas adhesivas se colocan en las residencias para recoger *Aedes* semanalmente, tras lo cual se detecta el antígeno

NS1 del dengue en dichos mosquitos. Cuando se detecta un mosquito con resultado positivo para dengue, se informa a la comunidad para que ejecute medidas de protección y de búsqueda y eliminación de vectores. El resultado principal se refiere al cambio porcentual en el (i) número de casos de dengue y (ii) en la duración de los brotes de dengue. Mientras que las otras medidas de resultado incluyen el cambio en el umbral de densidad de *Aedes* y cambios en los conocimientos, actitudes y prácticas relacionadas con el dengue de los grupos de población.

Análisis: esta es una vigilancia proactiva y precoz del dengue en el mosquito vector que no se basa en la notificación de los casos de dengue. La vigilancia con ovitrampas adhesivas debe poder ofrecer suficiente cobertura de manera eficaz para viviendas de varias plantas donde es posible que la población por unidad de superficie sea mayor. Además, atrapar mosquitos infectados por el dengue usando ovitrampas adhesivas ayuda a detener la transmisión del dengue que transporta el mosquito. Se prevé que los resultados de este ensayo aleatorio controlado ofrezcan una nueva herramienta de vigilancia proactiva, económica y precisa para la prevención y el control de los brotes de dengue.

Translated from English version into Spanish by Carmen Blazquez, Revised by Mayra León, through

TRANSLATORS
WITHOUT BORDERS
